# Synthesis and crystal structure of 2-(2,4-dioxo-6-methyl­pyran-3-yl­idene)-4-(4-hy­droxy­phen­yl)-2,3,4,5-tetra­hydro-1*H*-1,5-benzodiazepine

**DOI:** 10.1107/S2056989025003032

**Published:** 2025-04-08

**Authors:** Imane Faraj, Lhoussaine El Ghayati, Olivier Blacque, Tuncer Hökelek, Ahmed Mazzah, El Mokhtar Essassi, Nada Kheira Sebbar

**Affiliations:** a Laboratory of Heterocyclic Organic Chemistry Medicines Science Research Center Pharmacochemistry Competence Center Mohammed V University in Rabat Faculté des Sciences Av Ibn Battouta BP 1014 Rabat Morocco; b University of Zurich, Department of Chemistry, Winterthurerstrasse 190, CH-8057 Zurich, Switzerland; c Department of Physics, Hacettepe University, 06800 Beytepe, Ankara, Türkiye; d Science and Technology of Lille USR 3290, Villeneuve d’ascq cedex, France; e Laboratory of Organic and Physical Chemistry, Applied Bioorganic Chemistry Team, Faculty of Sciences, Ibnou Zohr University, Agadir, Morocco; f Laboratory of Plant Chemistry Organic and Bioorganic Synthesis Faculty of Sciences Mohammed V University in Rabat 4 Avenue Ibn Battouta BP 1014 RP Rabat Morocco; University of Aberdeen, United Kingdom

**Keywords:** crystal structure, benzodiazepine, C—H⋯π inter­action, hydrogen bond

## Abstract

The title compound, C_21_H_18_N_2_O_4_, contains non-planar diazepine (in a boat–sofa conformation) and pyran (envelope) rings. In the crystal, O—H⋯O and N—H⋯O hydrogen bonds link the mol­ecules, enclosing *R*_2_^2^(16) and *R*_2_^2^(24) ring motifs, to generate [110] chains. Very weak π–π stacking inter­actions between the phenyl rings of adjacent mol­ecules with an inter-centroid distance of 4.0264 (9) Å help to consolidate a three-dimensional architecture. The Hirshfeld surface analysis of the crystal structure indicates that the most important contributions for the crystal packing are from H⋯H (45.1%), H⋯O/O⋯H (23.2%) and H⋯C/C⋯H (19.2%) inter­actions.

## Abstract 2

In the crystal, O—H⋯O and N—H⋯O hydrogen bonds link the mol­ecules, enclosing *R*_2_^2^(16) and *R*_2_^2^(24) ring motifs, to generate [110] chains. Very weak π–π stacking inter­actions between the phenyl rings of adjacent mol­ecules help to consolidate a three-dimensional architecture.

## 1. Chemical context

1,5-Benzodiazepine derivatives are known for their potent biological activities, being used as anti­tubercular agents (Singh *et al.*, 2017 ▸), anti­convulsants (Jyoti & Mithlesh, 2013 ▸), anti­cancer agents (Gawandi *et al.*, 2021 ▸), anti­microbials (An *et al.*, 2016 ▸), and anti­depressants (Sharma *et al.*, 2017 ▸). This study continues our investigation into 1,5-benzodiazepine derivatives, as published by our team in earlier works (El Ghayati *et al.*, 2021 ▸; Essaghouani *et al.*, 2017 ▸). In this context, we synthesized the title compound, C_21_H_18_N_2_O_4_, (**I**), through the condensation reaction of the inter­mediate 3-[1-(2-amino-phenyl­imino)-eth­yl]-4-hy­droxy-6-methyl-pyran-2-one with 4-hy­droxy­benzalde­hyde in ethanol. In this report, we present the synthesis, mol­ecular and crystal structures and Hirshfeld surface analysis of (**I**).
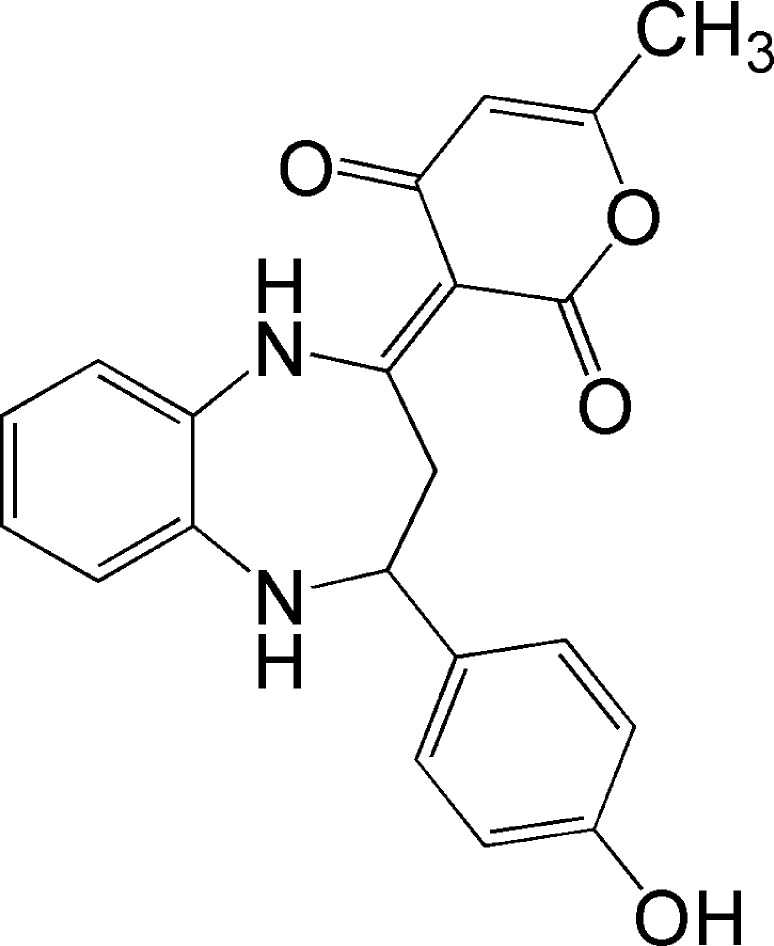


## 2. Structural commentary

**Figure 1:**
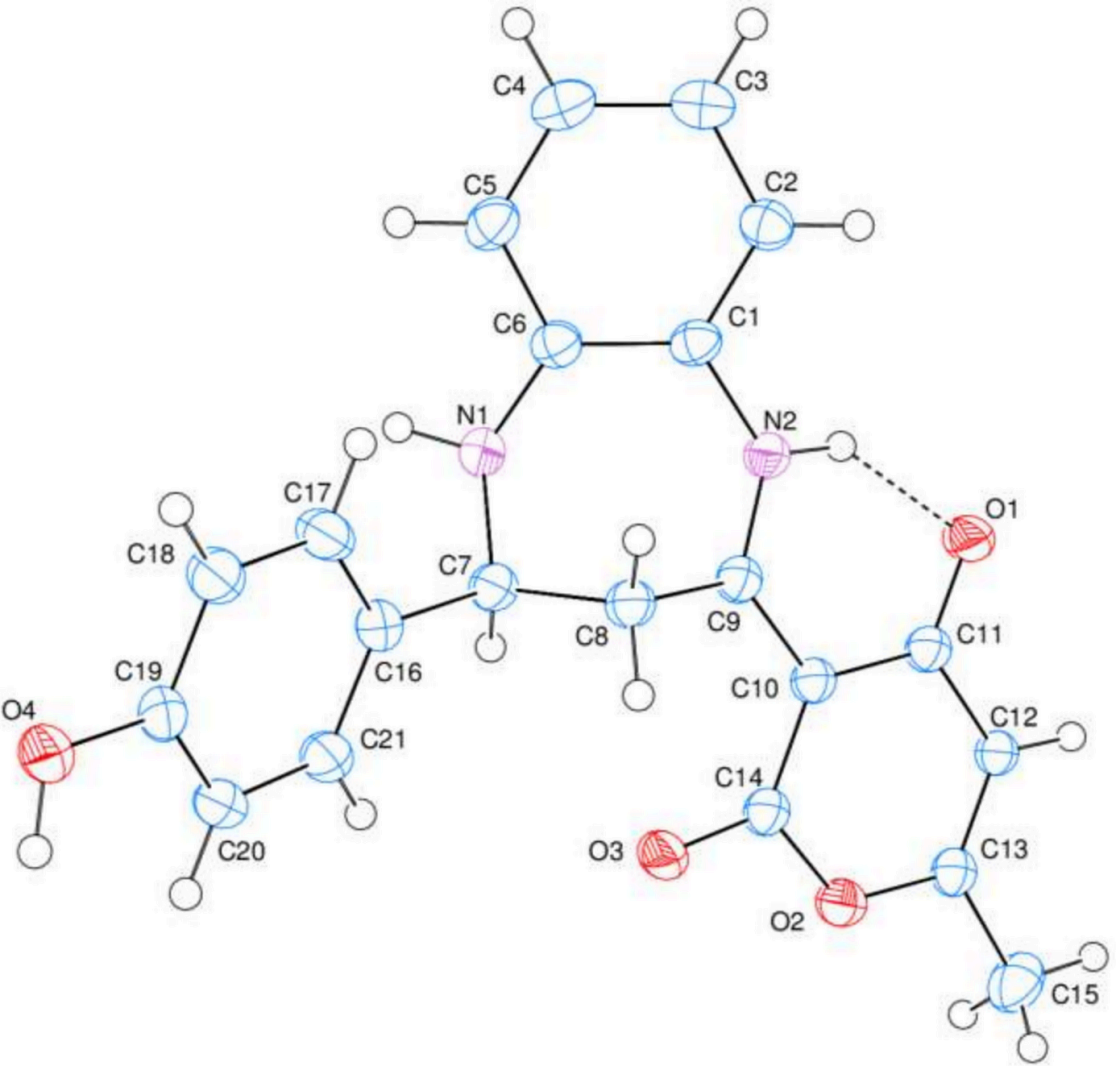
The mol­ecular structure of (**I**) showing 50% probability ellipsoids.

Compound (**I**) contains a benzodiazepine ring system besides pyran and phenyl rings (Fig. 1 ▸). The benzene (*A*, C1–C6) and phenyl (*C*, C16–C21) rings are oriented at a dihedral angle of 42.68 (5)°, and atom O4 is displaced by 0.0246 (12) Å from the mean plane of the *C* ring. The seven-membered diazepine ring (*B*, N1/N2/C1/C6–C9) is in a boat–sofa conformation (Boessenkool & Boeyens, 1980 ▸) with puckering amplitudes *Q*_T_ = 0.9082 (14) Å, *q*_2_ = 0.8845 (14) Å and *q*_3_ = 0.2062 (14) Å: atoms N1, N2, C7 and C9 form the base, C8 the prow and C1 and C6 the stern. On the other hand, the pyran, (*D*, O2/C10–C14), ring is in a shallow envelope conformation with puckering parameters *Q*_T_ = 0.0661 (14) Å, θ = 53 (1)° and φ = 5 (2)°. Atom C11 at the flap position is displaced by 0.0802 (14) Å from the best least-squares plane of the other five atoms. An intra­molecular N2—H2⋯O1 hydrogen bond (Table 1 ▸) between the diazepine and pyran rings completes an *S*(6) ring motif. Otherwise there are no unusual bond distances or inter­bond angles in the mol­ecule.

## 3. Supra­molecular features

**Figure 2:**
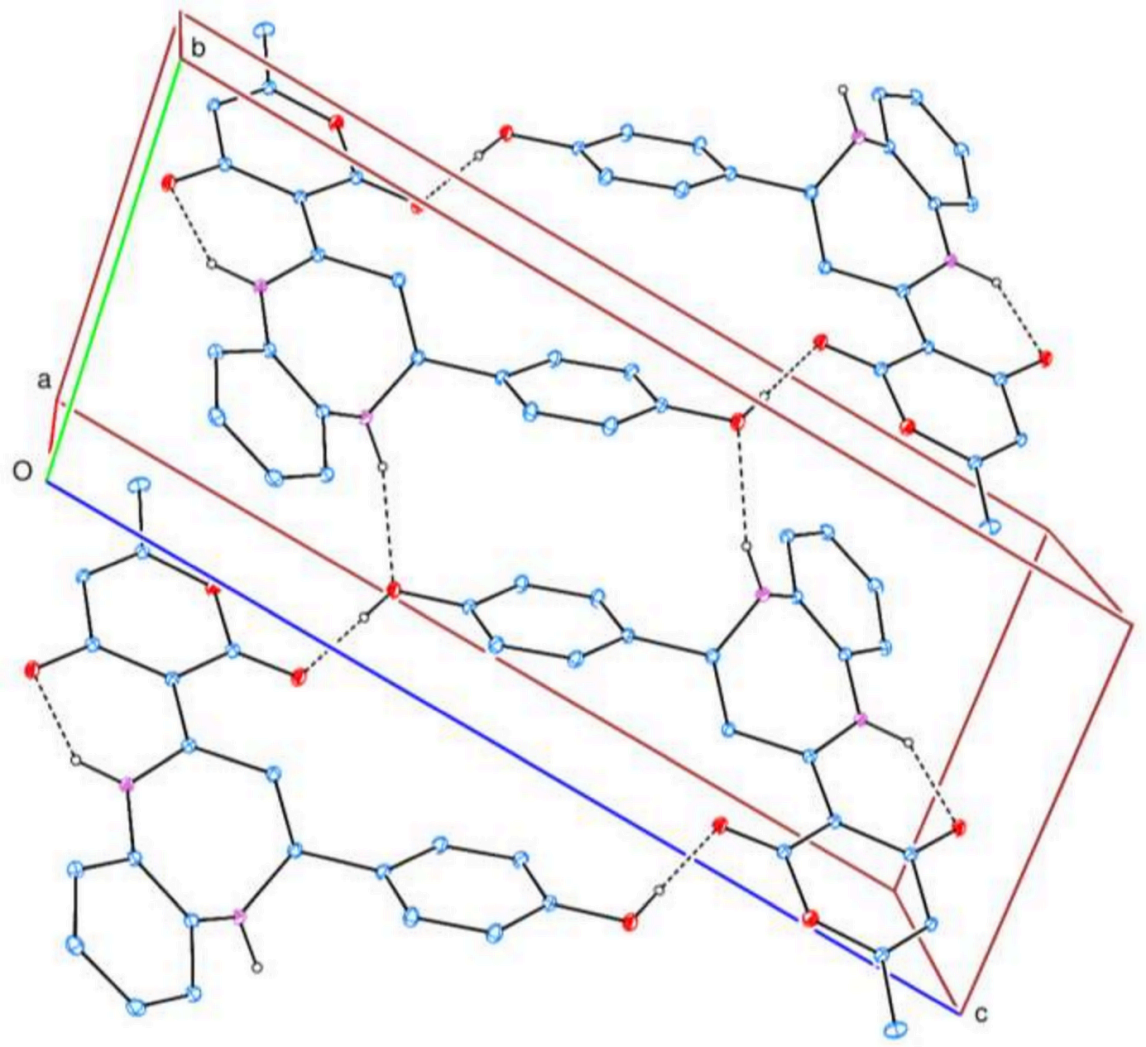
A partial packing diagram of (**I**) viewed down the *a*-axis direction. The inter­molecular O—H⋯O and N—H⋯O and intra­molecular N—H⋯O hydrogen bonds are shown as dashed lines. The other hydrogen atoms have been omitted for clarity.

In the crystal, O4—H4⋯O3 and N1—H1⋯O4 hydrogen bonds link the mol­ecules (Fig. 2 ▸), enclosing 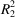
(16) and 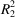
(24) ring motifs (Etter *et al.*, 1990 ▸). A [110] infinite chain results. A very weak π–π stacking inter­action between the *C* rings of adjacent mol­ecules with an inter-centroid distance of 4.0264 (9) Å may help to consolidate the three-dimensional architecture. There are no identified C—H⋯π(ring) inter­actions.

## 4. Hirshfeld surface analysis

**Figure 3:**
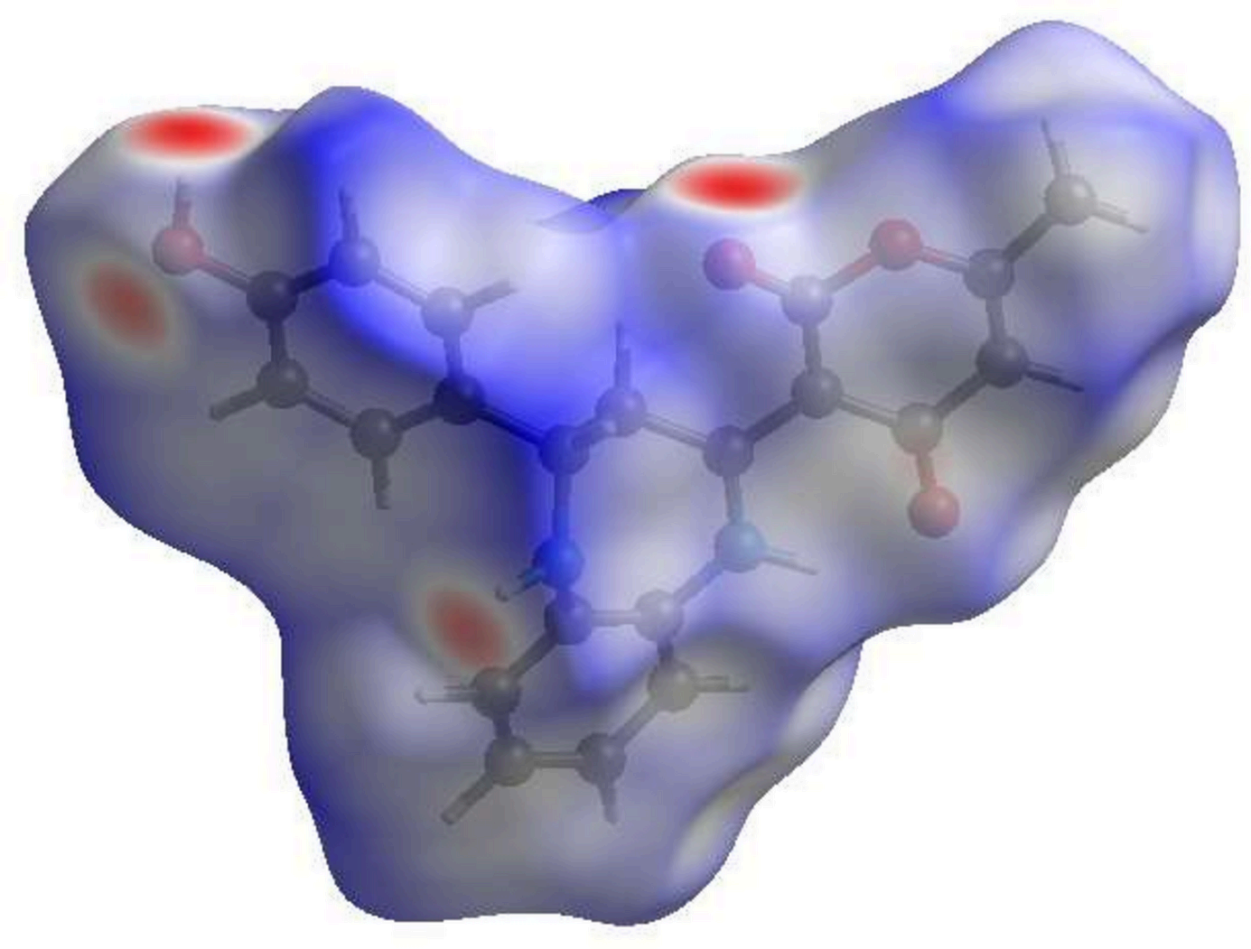
View of the three-dimensional Hirshfeld surface of (**I**) plotted over *d*_norm_.

**Figure 4:**
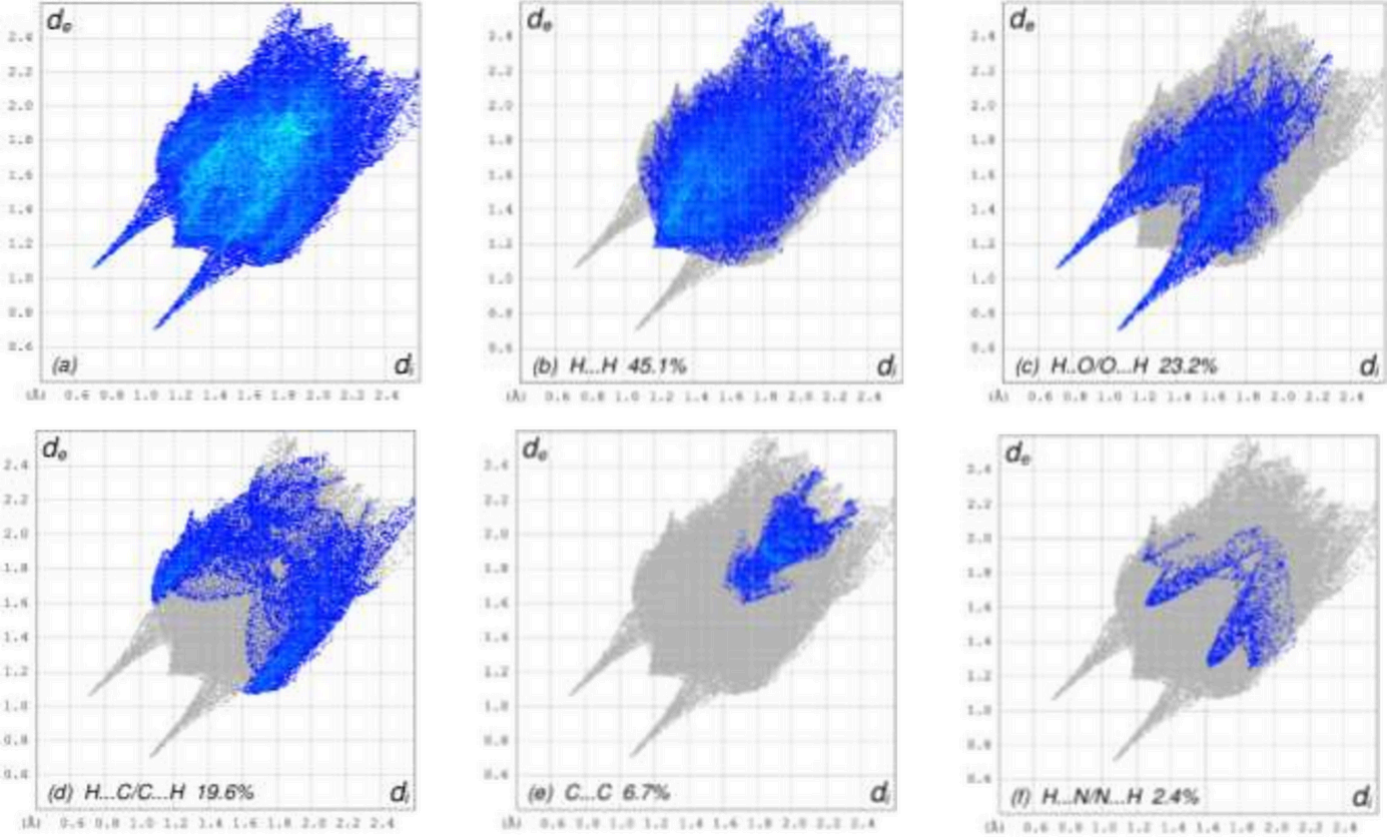
The two-dimensional fingerprint plots for (**I**), showing (*a*) all inter­actions, and delineated into (*b*) H⋯H, (*c*) H⋯O/O⋯H, (*d*) H⋯C/C⋯H, (*e*) C⋯C, (*f*) H⋯N/N⋯H inter­actions. The *d*_i_ and *d*_e_ values are the closest inter­nal and external distances (in Å) from given points on the Hirshfeld surface.

To visualize the inter­molecular inter­actions in the crystal of (**I**), a Hirshfeld surface (HS) analysis was carried out using *Crystal Explorer 17.5* (Spackman *et al.*, 2021 ▸). Fig. 3 ▸ shows the contact distances where the bright-red spots correspond to the respective donors and/or acceptors noted above. According to the two-dimensional fingerprint plots (McKinnon *et al.*, 2007 ▸), the H⋯H, H⋯O/O⋯H and H⋯C/C⋯H contacts make the most significant contributions to the HS, at 45.1%, 23.2% and 19.2%, respectively (Fig. 4 ▸).

## 5. Database survey

A search of the Cambridge Structural Database (CSD up­dated to January 2025; Groom *et al.*, 2016 ▸) for 2,3,4,5-tetrahydro-1*H*-benzo[*b*][1,4]diazepines substituted at the 2- and 4-positions gave a substantial number of hits, with seven deemed closely similar to the title molecule. These are **A** (Siddiqui & Siddiqui, 2020 ▸), compound **B** with *R* = thiophene and 4-ClC_6_H_4_, and *R*′ = 6-methyl-2*H*-pyran-2,4(3*H*)-dione, as well as *R* = 6-methyl-2*H*-pyran-2,4(3*H*)-dione and *R*′ = 3-BrC_6_H_4_ (Faidallah *et al.*, 2015 ▸), and compounds **C** (Wu & Wang, 2020 ▸) and **D** (Lal *et al.*, 2013 ▸) (see scheme below[Chem scheme2]). All have the tetrahydrodiazepine ring adopting a boat conformation, with total puckering amplitudes ranging from 0.702 (2) (for **A**) to 0.957 (2) Å (for **C**, *R* = thiophene). The dihedral angles between the mean plane of the benzo ring and those of the ring containing substituents on the seven-mem­bered ring vary considerably, likely due to packing considerations, as the steric bulk of these groups differs markedly.
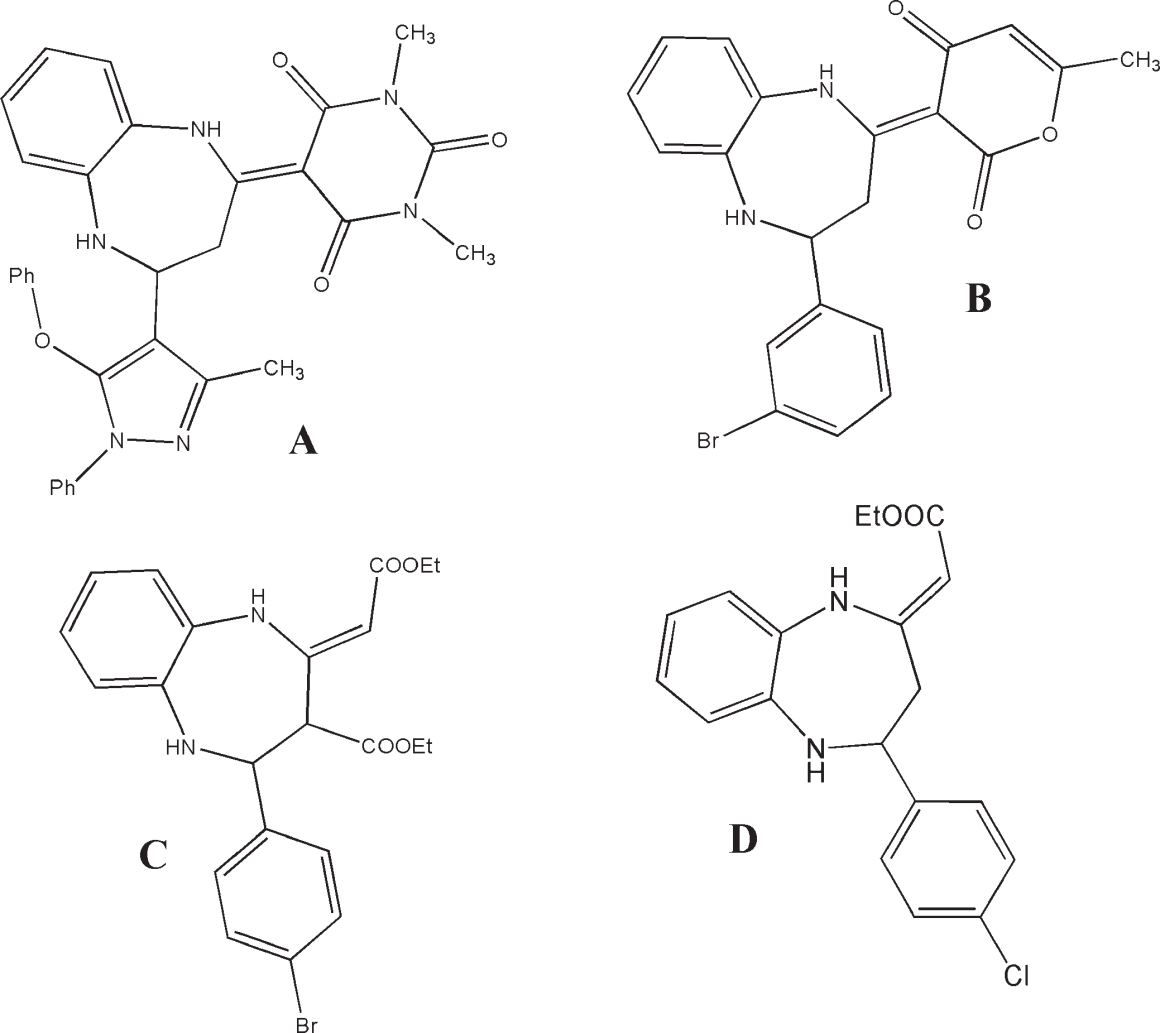


## 6. Synthesis and crystallization

A mixture of 3.87 mmol of 3-[1-(2-aminophenyl­imino)eth­yl]-4-hy­droxy-6-methyl-pyran-2-one in 40 ml of ethanol with 5.81 mmol of 4-hy­droxy­benzaldehyde, along with a catalytic amount of tri­fluoro­acetic acid was made up. The reaction mixture was refluxed for 4 h. After cooling and filtration, the formed precipitate was recrystallized from ethanol solution to obtain the title compound (**I**).

## 7. Refinement

Crystal data, data collection and structure refinement details are summarized in Table 2 ▸. The OH and NH hydrogen atoms were located in a difference-Fourier map and the positions were freely refined. The C-bound hydrogen-atom positions were calculated geometrically (C—H = 0.95–1.00 Å depending on hybridization) and refined using a riding model with *U*_iso_(H) = 1.2*U*_eq_(C) or 1.5*U*_eq_(methyl C).

## Supplementary Material

Crystal structure: contains datablock(s) I. DOI: 10.1107/S2056989025003032/hb8131sup1.cif

Supporting information file. DOI: 10.1107/S2056989025003032/hb8131Isup4.cdx

Supporting information file. DOI: 10.1107/S2056989025003032/hb8131Isup5.cml

Structure factors: contains datablock(s) I. DOI: 10.1107/S2056989025003032/hb8131Isup5.hkl

Supporting information file. DOI: 10.1107/S2056989025003032/hb8131sup3.txt

CCDC reference: 2440877

Additional supporting information:  crystallographic information; 3D view; checkCIF report

Additional supporting information:  crystallographic information; 3D view; checkCIF report

## Figures and Tables

**Table 1:** Hydrogen-bond geometry (Å, °)

*D*—H⋯*A*	*D*—H	H⋯*A*	*D*⋯*A*	*D*—H⋯*A*
O4—H4⋯O3^iii^	0.90 (3)	1.86 (3)	2.7597 (15)	178 (2)
N1—H1⋯O4^iv^	0.92 (2)	2.21 (2)	3.1028 (16)	165.2 (17)
N2—H2⋯O1	0.93 (2)	1.80 (2)	2.5882 (15)	140.8 (18)

Symmetry codes: (iii) 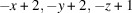; (iv) 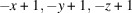.

**Table 2:** Experimental details

Crystal data	
Chemical formula	C_21_H_18_N_2_O_4_
*M*_r_	362.37
Crystal system, space group	Triclinic, *P* 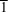
Temperature (K)	160
*a*, *b*, *c* (Å)	6.3757 (1), 7.7506 (1), 17.9997 (4)
α, β, γ (°)	100.967 (2), 97.373 (2), 100.127 (2)
*V* (Å^3^)	847.49 (3)
*Z*	2
Radiation type	Cu *K*α
μ (mm^−1^)	0.82
Crystal size (mm)	0.10 × 0.03 × 0.02

Data collection	
Diffractometer	XtaLAB Synergy, Dualflex, HyPix
Absorption correction	Analytical (*CrysAlis PRO*; Rigaku OD, 2024 ▸)
*T*_min_, *T*_max_	0.941, 0.988
No. of measured, independent and observed [*I* > 2σ(*I*)] reflections	18745, 3608, 3159
*R*_int_	0.028
(sin θ/λ)_max_ (Å^−1^)	0.638

Refinement	
*R*[*F*^2^ > 2σ(*F*^2^)], *wR*(*F*^2^), *S*	0.041, 0.115, 1.08
No. of reflections	3608
No. of parameters	258
H-atom treatment	H atoms treated by a mixture of independent and constrained refinement
Δρ_max_, Δρ_min_ (e Å^−3^)	0.45, −0.32

Computer programs: *CrysAlis PRO* (Rigaku OD, 2024 ▸), *SHELXT* (Sheldrick, 2015*a* ▸), *SHELXL* (Sheldrick, 2015*b* ▸) and *OLEX2* (Dolomanov *et al.*, 2009 ▸).

## supplementary crystallographic information

### 4-(4-Hydroxyphenyl)-2-(6-methyl-2,4-dioxopyran-3-ylidene)-2,3,4,5-tetrahydro-1H-1,5-benzodiazepine Crystal data

**Table:**

C_21_H_18_N_2_O_4_	*Z* = 2
*M**_r_* = 362.37	*F*(000) = 380
Triclinic, *P*1	*D*_x_ = 1.420 Mg m^−^^3^
*a* = 6.3757 (1) Å	Cu *K*α radiation, λ = 1.54184 Å
*b* = 7.7506 (1) Å	Cell parameters from 9714 reflections
*c* = 17.9997 (4) Å	θ = 2.5–79.1°
α = 100.967 (2)°	µ = 0.82 mm^−^^1^
β = 97.373 (2)°	*T* = 160 K
γ = 100.127 (2)°	Needle, yellow
*V* = 847.49 (3) Å^3^	0.10 × 0.03 × 0.02 mm

### 4-(4-Hydroxyphenyl)-2-(6-methyl-2,4-dioxopyran-3-ylidene)-2,3,4,5-tetrahydro-1H-1,5-benzodiazepine Data collection

**Table:**

XtaLAB Synergy, Dualflex, HyPix diffractometer	3608 independent reflections
Radiation source: micro-focus sealed X-ray tube, PhotonJet (Cu) X-ray Source	3159 reflections with *I* > 2σ(*I*)
Mirror monochromator	*R*_int_ = 0.028
Detector resolution: 10.0000 pixels mm^-1^	θ_max_ = 79.4°, θ_min_ = 2.5°
ω scans	*h* = −8→8
Absorption correction: analytical (CrysAlisPro; Rigaku OD, 2024)	*k* = −9→7
*T*_min_ = 0.941, *T*_max_ = 0.988	*l* = −21→22
18745 measured reflections	

### 4-(4-Hydroxyphenyl)-2-(6-methyl-2,4-dioxopyran-3-ylidene)-2,3,4,5-tetrahydro-1H-1,5-benzodiazepine Refinement

**Table:**

Refinement on *F*^2^	Hydrogen site location: mixed
Least-squares matrix: full	H atoms treated by a mixture of independent and constrained refinement
*R*[*F*^2^ > 2σ(*F*^2^)] = 0.041	*w* = 1/[σ^2^(*F*_o_^2^) + (0.057*P*)^2^ + 0.3065*P*] where *P* = (*F*_o_^2^ + 2*F*_c_^2^)/3
*wR*(*F*^2^) = 0.115	(Δ/σ)_max_ < 0.001
*S* = 1.08	Δρ_max_ = 0.45 e Å^−^^3^
3608 reflections	Δρ_min_ = −0.31 e Å^−^^3^
258 parameters	Extinction correction: SHELXL (Sheldrick, 2015b), Fc^*^=kFc[1+0.001xFc^2^λ^3^/sin(2θ)]^-1/4^
0 restraints	Extinction coefficient: 0.0020 (5)
Primary atom site location: dual	

### 4-(4-Hydroxyphenyl)-2-(6-methyl-2,4-dioxopyran-3-ylidene)-2,3,4,5-tetrahydro-1H-1,5-benzodiazepine Special details

**Table:**

Geometry. All esds (except the esd in the dihedral angle between two l.s. planes) are estimated using the full covariance matrix. The cell esds are taken into account individually in the estimation of esds in distances, angles and torsion angles; correlations between esds in cell parameters are only used when they are defined by crystal symmetry. An approximate (isotropic) treatment of cell esds is used for estimating esds involving l.s. planes.

### 4-(4-Hydroxyphenyl)-2-(6-methyl-2,4-dioxopyran-3-ylidene)-2,3,4,5-tetrahydro-1H-1,5-benzodiazepine Fractional atomic coordinates and isotropic or equivalent isotropic displacement parameters (Å^2^)

**Table:**

	*x*	*y*	*z*	*U*_iso_*/*U*_eq_	
O1	0.65055 (17)	0.66657 (15)	0.03539 (6)	0.0334 (3)	
O2	1.17867 (16)	0.94555 (14)	0.18839 (6)	0.0314 (2)	
O3	1.03777 (17)	0.89041 (15)	0.28886 (6)	0.0352 (3)	
O4	0.58361 (18)	0.87903 (15)	0.63441 (6)	0.0335 (3)	
H4	0.709 (4)	0.952 (3)	0.6591 (14)	0.060 (7)*	
N1	0.47933 (19)	0.44213 (16)	0.28508 (7)	0.0283 (3)	
H1	0.462 (3)	0.363 (3)	0.3168 (11)	0.041 (5)*	
N2	0.46259 (18)	0.58760 (16)	0.14729 (7)	0.0257 (3)	
H2	0.478 (3)	0.574 (3)	0.0958 (12)	0.044 (5)*	
C1	0.2753 (2)	0.49401 (18)	0.16861 (8)	0.0255 (3)	
C2	0.0839 (2)	0.45358 (19)	0.11617 (8)	0.0289 (3)	
H2A	0.081763	0.494087	0.069522	0.035*	
C3	−0.1035 (2)	0.3553 (2)	0.13089 (9)	0.0330 (3)	
H3	−0.232941	0.326686	0.094441	0.040*	
C4	−0.0992 (2)	0.2990 (2)	0.19981 (9)	0.0337 (3)	
H4A	−0.227750	0.234648	0.211340	0.040*	
C5	0.0913 (2)	0.33631 (19)	0.25171 (8)	0.0306 (3)	
H5	0.091044	0.296638	0.298496	0.037*	
C6	0.2848 (2)	0.43108 (18)	0.23716 (8)	0.0257 (3)	
C7	0.6316 (2)	0.61210 (19)	0.32150 (8)	0.0272 (3)	
H7	0.781572	0.592843	0.318419	0.033*	
C8	0.5909 (2)	0.75522 (18)	0.27661 (8)	0.0266 (3)	
H8A	0.441204	0.773084	0.277664	0.032*	
H8B	0.690582	0.870450	0.301855	0.032*	
C9	0.6225 (2)	0.70452 (17)	0.19521 (7)	0.0246 (3)	
C10	0.8083 (2)	0.78109 (18)	0.16665 (7)	0.0247 (3)	
C11	0.8057 (2)	0.75700 (18)	0.08500 (8)	0.0265 (3)	
C12	0.9941 (2)	0.84607 (18)	0.06080 (7)	0.0252 (3)	
H12	0.994265	0.840831	0.007649	0.030*	
C13	1.1697 (2)	0.93642 (18)	0.11166 (8)	0.0270 (3)	
C14	1.0025 (2)	0.87205 (18)	0.21893 (8)	0.0266 (3)	
C15	1.3704 (3)	1.0348 (2)	0.09359 (11)	0.0425 (4)	
H15A	1.398618	1.160599	0.121192	0.064*	
H15B	1.354219	1.028990	0.038227	0.064*	
H15C	1.491521	0.980219	0.109353	0.064*	
C16	0.6170 (2)	0.67727 (19)	0.40544 (8)	0.0281 (3)	
C17	0.4208 (2)	0.6658 (2)	0.43198 (8)	0.0337 (3)	
H17	0.290729	0.610431	0.397254	0.040*	
C18	0.4116 (2)	0.7339 (2)	0.50830 (8)	0.0340 (3)	
H18	0.276168	0.724100	0.525576	0.041*	
C19	0.6007 (2)	0.81657 (19)	0.55939 (8)	0.0282 (3)	
C20	0.7980 (2)	0.8286 (2)	0.53405 (8)	0.0336 (3)	
H20	0.927986	0.884150	0.568830	0.040*	
C21	0.8048 (2)	0.7590 (2)	0.45751 (8)	0.0328 (3)	
H21	0.940413	0.767471	0.440438	0.039*	

### 4-(4-Hydroxyphenyl)-2-(6-methyl-2,4-dioxopyran-3-ylidene)-2,3,4,5-tetrahydro-1H-1,5-benzodiazepine Atomic displacement parameters (Å^2^)

**Table:**

	*U*^11^	*U*^22^	*U*^33^	*U*^12^	*U*^13^	*U*^23^
O1	0.0320 (5)	0.0402 (6)	0.0219 (5)	−0.0044 (4)	0.0028 (4)	0.0037 (4)
O2	0.0288 (5)	0.0357 (5)	0.0268 (5)	0.0007 (4)	0.0040 (4)	0.0062 (4)
O3	0.0316 (5)	0.0468 (6)	0.0216 (5)	−0.0040 (4)	0.0018 (4)	0.0071 (4)
O4	0.0352 (6)	0.0388 (6)	0.0225 (5)	0.0007 (5)	0.0044 (4)	0.0037 (4)
N1	0.0298 (6)	0.0288 (6)	0.0258 (6)	0.0023 (5)	0.0034 (5)	0.0085 (5)
N2	0.0250 (6)	0.0293 (6)	0.0215 (6)	0.0012 (4)	0.0054 (4)	0.0051 (4)
C1	0.0242 (6)	0.0252 (6)	0.0258 (6)	0.0025 (5)	0.0069 (5)	0.0025 (5)
C2	0.0285 (7)	0.0291 (7)	0.0274 (7)	0.0052 (5)	0.0036 (5)	0.0034 (5)
C3	0.0251 (7)	0.0324 (7)	0.0368 (8)	0.0031 (6)	0.0024 (6)	0.0006 (6)
C4	0.0275 (7)	0.0308 (7)	0.0407 (8)	0.0011 (6)	0.0114 (6)	0.0035 (6)
C5	0.0322 (7)	0.0296 (7)	0.0294 (7)	0.0023 (6)	0.0105 (6)	0.0054 (5)
C6	0.0260 (6)	0.0243 (6)	0.0251 (6)	0.0032 (5)	0.0056 (5)	0.0022 (5)
C7	0.0253 (6)	0.0313 (7)	0.0241 (6)	0.0026 (5)	0.0055 (5)	0.0056 (5)
C8	0.0265 (6)	0.0284 (7)	0.0231 (6)	0.0016 (5)	0.0061 (5)	0.0034 (5)
C9	0.0257 (6)	0.0247 (6)	0.0233 (6)	0.0037 (5)	0.0047 (5)	0.0058 (5)
C10	0.0258 (7)	0.0257 (6)	0.0217 (6)	0.0024 (5)	0.0049 (5)	0.0048 (5)
C11	0.0276 (7)	0.0268 (6)	0.0237 (6)	0.0020 (5)	0.0048 (5)	0.0053 (5)
C12	0.0257 (6)	0.0303 (7)	0.0184 (6)	0.0006 (5)	0.0064 (5)	0.0056 (5)
C13	0.0284 (7)	0.0287 (7)	0.0244 (6)	0.0046 (5)	0.0064 (5)	0.0072 (5)
C14	0.0276 (7)	0.0278 (7)	0.0237 (6)	0.0025 (5)	0.0060 (5)	0.0061 (5)
C15	0.0309 (8)	0.0484 (9)	0.0552 (10)	0.0073 (7)	0.0146 (7)	0.0243 (8)
C16	0.0287 (7)	0.0316 (7)	0.0233 (6)	0.0034 (5)	0.0038 (5)	0.0076 (5)
C17	0.0258 (7)	0.0455 (8)	0.0252 (7)	0.0023 (6)	0.0010 (5)	0.0030 (6)
C18	0.0266 (7)	0.0464 (9)	0.0271 (7)	0.0037 (6)	0.0056 (5)	0.0066 (6)
C19	0.0326 (7)	0.0290 (7)	0.0219 (6)	0.0030 (5)	0.0037 (5)	0.0066 (5)
C20	0.0286 (7)	0.0413 (8)	0.0255 (7)	−0.0035 (6)	0.0004 (5)	0.0062 (6)
C21	0.0261 (7)	0.0427 (8)	0.0269 (7)	−0.0001 (6)	0.0047 (5)	0.0078 (6)

### 4-(4-Hydroxyphenyl)-2-(6-methyl-2,4-dioxopyran-3-ylidene)-2,3,4,5-tetrahydro-1H-1,5-benzodiazepine Geometric parameters (Å, º)

**Table:**

O1—C11	1.2484 (17)	C7—C16	1.5195 (18)
O2—C13	1.3626 (16)	C8—H8A	0.9900
O2—C14	1.3933 (16)	C8—H8B	0.9900
O3—C14	1.2260 (17)	C8—C9	1.4922 (18)
O4—H4	0.90 (3)	C9—C10	1.4300 (18)
O4—C19	1.3702 (17)	C10—C11	1.4434 (18)
N1—H1	0.92 (2)	C10—C14	1.4341 (19)
N1—C6	1.3963 (18)	C11—C12	1.4375 (18)
N1—C7	1.4717 (18)	C12—H12	0.9500
N2—H2	0.93 (2)	C12—C13	1.3414 (19)
N2—C1	1.4206 (17)	C13—C15	1.478 (2)
N2—C9	1.3254 (17)	C15—H15A	0.9800
C1—C2	1.3914 (19)	C15—H15B	0.9800
C1—C6	1.4086 (19)	C15—H15C	0.9800
C2—H2A	0.9500	C16—C17	1.390 (2)
C2—C3	1.384 (2)	C16—C21	1.390 (2)
C3—H3	0.9500	C17—H17	0.9500
C3—C4	1.390 (2)	C17—C18	1.387 (2)
C4—H4A	0.9500	C18—H18	0.9500
C4—C5	1.384 (2)	C18—C19	1.389 (2)
C5—H5	0.9500	C19—C20	1.386 (2)
C5—C6	1.4027 (19)	C20—H20	0.9500
C7—H7	1.0000	C20—C21	1.390 (2)
C7—C8	1.5295 (19)	C21—H21	0.9500
			
O1···N2	2.5882 (16)	C3···C10^v^	3.400 (2)
O1···O1^i^	2.8900 (16)	C1···H15A^vi^	2.84
O2···C5^ii^	3.1976 (18)	C1···H8A	2.59
O3···C8	2.8223 (17)	C2···H12^i^	2.79
O3···O4^iii^	2.7598 (16)	C6···H8A	2.59
O1···H2	1.80 (2)	C11···H2	2.36 (2)
O1···H2^i^	2.65 (2)	C14···H8B	2.64
O2···H4^iii^	2.67 (2)	C14···H4^iii^	2.65 (2)
O3···H8B	2.24	C17···H1	2.90 (2)
O3···H4^iii^	1.86 (3)	C19···H1^iv^	2.88 (2)
O4···H1^iv^	2.21 (2)	H1···H5	2.2962
N1···N2	2.9107 (17)	H2···H2A	2.45
N1···H17	2.72	H4···H20	2.32
C2···C11^v^	3.272 (2)	H7···H21	2.35
C2···C10^v^	3.3849 (19)		
			
C13—O2—C14	122.21 (11)	C10—C9—C8	123.66 (12)
C19—O4—H4	109.6 (16)	C9—C10—C11	120.11 (12)
C6—N1—H1	110.6 (12)	C9—C10—C14	120.03 (12)
C6—N1—C7	123.76 (12)	C14—C10—C11	119.74 (12)
C7—N1—H1	113.6 (12)	O1—C11—C10	124.06 (12)
C1—N2—H2	120.6 (13)	O1—C11—C12	119.13 (12)
C9—N2—H2	114.0 (13)	C12—C11—C10	116.80 (12)
C9—N2—C1	125.33 (11)	C11—C12—H12	119.3
C2—C1—N2	117.37 (12)	C13—C12—C11	121.42 (12)
C2—C1—C6	120.69 (12)	C13—C12—H12	119.3
C6—C1—N2	121.73 (12)	O2—C13—C15	112.37 (13)
C1—C2—H2A	119.5	C12—C13—O2	121.51 (12)
C3—C2—C1	121.03 (13)	C12—C13—C15	126.11 (13)
C3—C2—H2A	119.5	O2—C14—C10	117.90 (11)
C2—C3—H3	120.5	O3—C14—O2	114.04 (12)
C2—C3—C4	118.99 (14)	O3—C14—C10	128.03 (13)
C4—C3—H3	120.5	C13—C15—H15A	109.5
C3—C4—H4A	119.9	C13—C15—H15B	109.5
C5—C4—C3	120.29 (13)	C13—C15—H15C	109.5
C5—C4—H4A	119.9	H15A—C15—H15B	109.5
C4—C5—H5	119.1	H15A—C15—H15C	109.5
C4—C5—C6	121.83 (13)	H15B—C15—H15C	109.5
C6—C5—H5	119.1	C17—C16—C7	122.38 (12)
N1—C6—C1	122.60 (12)	C17—C16—C21	118.19 (13)
N1—C6—C5	119.93 (12)	C21—C16—C7	119.36 (12)
C5—C6—C1	117.06 (13)	C16—C17—H17	119.4
N1—C7—H7	108.1	C18—C17—C16	121.16 (13)
N1—C7—C8	109.11 (11)	C18—C17—H17	119.4
N1—C7—C16	113.14 (11)	C17—C18—H18	120.1
C8—C7—H7	108.1	C17—C18—C19	119.86 (14)
C16—C7—H7	108.1	C19—C18—H18	120.1
C16—C7—C8	110.02 (11)	O4—C19—C18	117.69 (13)
C7—C8—H8A	109.2	O4—C19—C20	122.47 (13)
C7—C8—H8B	109.2	C20—C19—C18	119.81 (13)
H8A—C8—H8B	107.9	C19—C20—H20	120.2
C9—C8—C7	112.11 (11)	C19—C20—C21	119.66 (13)
C9—C8—H8A	109.2	C21—C20—H20	120.2
C9—C8—H8B	109.2	C16—C21—H21	119.3
N2—C9—C8	116.72 (12)	C20—C21—C16	121.32 (14)
N2—C9—C10	119.55 (12)	C20—C21—H21	119.3
			
O1—C11—C12—C13	−176.20 (14)	C8—C7—C16—C21	−95.22 (15)
O4—C19—C20—C21	178.55 (14)	C8—C9—C10—C11	−165.74 (13)
N1—C7—C8—C9	−60.37 (14)	C8—C9—C10—C14	18.3 (2)
N1—C7—C16—C17	−40.57 (19)	C9—N2—C1—C2	147.66 (14)
N1—C7—C16—C21	142.47 (14)	C9—N2—C1—C6	−37.4 (2)
N2—C1—C2—C3	176.89 (12)	C9—C10—C11—O1	−2.4 (2)
N2—C1—C6—N1	−5.7 (2)	C9—C10—C11—C12	176.74 (12)
N2—C1—C6—C5	−178.41 (12)	C9—C10—C14—O2	−179.54 (12)
N2—C9—C10—C11	11.1 (2)	C9—C10—C14—O3	2.7 (2)
N2—C9—C10—C14	−164.86 (13)	C10—C11—C12—C13	4.6 (2)
C1—N2—C9—C8	−6.8 (2)	C11—C10—C14—O2	4.5 (2)
C1—N2—C9—C10	176.18 (12)	C11—C10—C14—O3	−173.28 (14)
C1—C2—C3—C4	1.0 (2)	C11—C12—C13—O2	1.0 (2)
C2—C1—C6—N1	169.01 (13)	C11—C12—C13—C15	−179.05 (14)
C2—C1—C6—C5	−3.7 (2)	C13—O2—C14—O3	179.40 (13)
C2—C3—C4—C5	−1.9 (2)	C13—O2—C14—C10	1.33 (19)
C3—C4—C5—C6	0.1 (2)	C14—O2—C13—C12	−4.2 (2)
C4—C5—C6—N1	−170.18 (13)	C14—O2—C13—C15	175.86 (12)
C4—C5—C6—C1	2.7 (2)	C14—C10—C11—O1	173.61 (13)
C6—N1—C7—C8	−21.39 (17)	C14—C10—C11—C12	−7.29 (19)
C6—N1—C7—C16	101.43 (15)	C16—C7—C8—C9	174.97 (11)
C6—C1—C2—C3	1.9 (2)	C16—C17—C18—C19	0.5 (2)
C7—N1—C6—C1	59.64 (19)	C17—C16—C21—C20	−0.3 (2)
C7—N1—C6—C5	−127.87 (14)	C17—C18—C19—O4	−178.94 (14)
C7—C8—C9—N2	78.98 (15)	C17—C18—C19—C20	−0.8 (2)
C7—C8—C9—C10	−104.10 (15)	C18—C19—C20—C21	0.5 (2)
C7—C16—C17—C18	−176.98 (14)	C19—C20—C21—C16	0.1 (2)
C7—C16—C21—C20	176.77 (14)	C21—C16—C17—C18	0.0 (2)
C8—C7—C16—C17	81.74 (17)		

Symmetry codes: (i) −*x*+1, −*y*+1, −*z*; (ii) *x*+1, *y*+1, *z*; (iii) −*x*+2, −*y*+2, −*z*+1; (iv) −*x*+1, −*y*+1, −*z*+1; (v) *x*−1, *y*, *z*; (vi) *x*−1, *y*−1, *z*.

### 4-(4-Hydroxyphenyl)-2-(6-methyl-2,4-dioxopyran-3-ylidene)-2,3,4,5-tetrahydro-1H-1,5-benzodiazepine Hydrogen-bond geometry (Å, º)

**Table:**

*D*—H···*A*	*D*—H	H···*A*	*D*···*A*	*D*—H···*A*
O4—H4···O3^iii^	0.90 (3)	1.86 (3)	2.7597 (15)	178 (2)
N1—H1···O4^iv^	0.92 (2)	2.21 (2)	3.1028 (16)	165.2 (17)
N2—H2···O1	0.93 (2)	1.80 (2)	2.5882 (15)	140.8 (18)

Symmetry codes: (iii) −*x*+2, −*y*+2, −*z*+1; (iv) −*x*+1, −*y*+1, −*z*+1.
